# Nitric Oxide Synthetic Pathway in Patients with Microvascular Angina and Its Relations with Oxidative Stress

**DOI:** 10.1155/2014/726539

**Published:** 2014-04-22

**Authors:** Benedetta Porro, Sonia Eligini, Fabrizio Veglia, Alessandro Lualdi, Isabella Squellerio, Susanna Fiorelli, Marta Giovannardi, Elisa Chiorino, Alessia Dalla Cia, Mauro Crisci, José Pablo Werba, Elena Tremoli, Viviana Cavalca

**Affiliations:** ^1^Centro Cardiologico Monzino, I.R.C.C.S., 20138 Milan, Italy; ^2^Dipartimento di Scienze Cliniche e di Comunità, Università degli Studi di Milano, 20138 Milan, Italy; ^3^Dipartimento di Scienze Farmacologiche e Biomolecolari, Università degli Studi di Milano, 20133 Milan, Italy

## Abstract

A decreased nitric oxide (NO) bioavailability and an increased oxidative stress play a pivotal role in different cardiovascular pathologies. As red blood cells (RBCs) participate in NO formation in the bloodstream, the aim of this study was to outline the metabolic profile of L-arginine (Arg)/NO pathway and of oxidative stress status in RBCs and in plasma of patients with microvascular angina (MVA), investigating similarities and differences with respect to coronary artery disease (CAD) patients or healthy controls (Ctrl). Analytes involved in Arg/NO pathway and the ratio of oxidized and reduced forms of glutathione were measured by LC-MS/MS. The arginase and the NO synthase (NOS) expression were evaluated by immunofluorescence staining. RBCs from MVA patients show increased levels of NO synthesis inhibitors, parallel to that found in plasma, and a reduction of NO synthase expression. When summary scores were computed, both patient groups were associated with a positive oxidative score and a negative NO score, with the CAD group located in a more extreme position with respect to Ctrl. This finding points out to an impairment of the capacity of RBCs to produce NO in a pathological condition characterized mostly by alterations at the microvascular bed with no significant coronary stenosis.

## 1. Introduction

NO is an important signaling molecule involved in the maintenance of vascular function. It promotes several beneficial effects in the vasculature by inducing vasorelaxation, inhibition of leukocyte-endothelium adhesion, smooth muscle cells migration and proliferation, and platelet aggregation [1, 2]. A decreased NO bioavailability is well documented in several cardiovascular diseases, including hypertension, atherosclerosis, and ischemia-reperfusion injury. A reduction of circulating NO species (nitrite and nitrosylated compounds), which contribute to the total NO availability, is described in individuals with endothelial dysfunction. The decrease is correlated with increasing numbers of cardiovascular risk factors [3–5].

NO is synthesized by the enzymatic action of NO synthases (NOSs), catalyzing the oxidation of the amino acid L-arginine (Arg) to equimolar amounts of NO and L-citrulline (Cit), in the presence of oxygen and cofactors. Although synthesis and release of NO are related to the substrate bioavailability [6], other potential causes of NO deficiency in disease settings have been proposed. Among these, the high circulating levels of endogenous methylarginines, that is, symmetric, asymmetric dimethylarginine (SDMA, ADMA) and monomethylarginine (MMA), act as NO-synthesis inhibitors [7, 8]. In addition, oxidative stress plays a pivotal role in determining NO bioavailability by the oxidation of the cofactors/the enzymes involved in NO metabolism or by the direct inactivation of NO.

Endothelial cells are considered the major source of NO in the vasculature; however, it has been shown that also circulating cells may contribute to NO synthesis, that is, platelets, monocytes, and red blood cells (RBCs). RBCs express functional NOS [9, 10], similar to the enzyme of endothelial cells [11], which serves as an intraluminal NO source and contributes to the regulation of systemic blood pressure [12]. In addition, the transporter for cationic amino acids [13] and all the enzymes involved in dimethylarginine metabolism (synthesis and catabolism) [14] have been identified in RBCs. Human RBCs also express the enzyme arginase that competes with NOS for their common substrate Arg to form L-ornithine (Orn) [15]. Two different isoforms of arginase are expressed in human [16] and, recently, it has been shown that arginase I plays an essential role in the control of RBC-NOS function and in the release of bioactive NO [17]. Indeed, in experimental models of atherosclerosis [18], myocardial ischemia [19], hypertension [20], and ageing [21], arginase activity has been reported to be upregulated at vascular level.

Microvascular angina (MVA) is a pathological condition characterized by the typical anginal pain, electrocardiographic (ECG) abnormalities at rest (ST-segment depression or T-wave inversion), all features that increase during exercise, in the presence of nonobstructed epicardial coronary arteries [22–24]. Even if the pathophysiology of MVA has not been disentangled yet, insulin resistance, abnormal autonomic control, enhanced sodium hydrogen exchange activity, abnormal cardiac sensitivity, and microvascular spasm have been proposed as potential causes [25]. In addition, increased concentrations of circulating C-reactive protein have been shown to correlate with vascular abnormalities in patients with MVA, suggesting a role of inflammation in this pathological condition [26].

Oxidative stress* per se*, either directly or through the reduction of NO bioavailability leading to an impairment of endothelium dependent vasodilation, has been involved in the pathophysiology of MVA [27, 28]. In particular, impaired endothelium-dependent vasodilatation of the coronary microvasculature [27] and its related impaired function, which limits coronary flow reserve [28–30], have been proposed to induce MVA syndrome.

Alterations in flow-mediated coronary dilation are a frequent finding in patients with MVA. In the microcirculation, blood flow is largely dependent on hemorheological properties, particularly RBC deformability, whose importance increases in capillaries compared with larger vessel [31]. A decreased RBC deformability has been shown in patients with CAD and diabetes mellitus [32] and it has been related to a decreased NO release [33]. In addition, due to the structural properties and blood flow in the microcirculatory bed, blood cells are in close contact with endothelium. As it has been shown that eNOS expression decreases in the microvasculature [34], it could be speculated that within capillaries RBC-NOS may play a more decisive role [35].

Moreover, it has been recently shown that in patients with cardiac syndrome X an increase of red cell distribution width (RDW), a measurement of size variability, and of erythrocytes, occurs [36]. Even if it has been reported that reduction of nitrate and nitrite, coupled to increases in ADMA and SDMA, occurs in plasma of MVA patients [37, 38], no information on the levels of the single components of the NO pathway in RBCs is available yet. Thus, in this study, we have characterized oxidative stress and the NO biosynthetic pathway in RBCs of MVA patients in comparison to patients with coronary artery disease (CAD) or healthy subjects (Ctrl).

## 2. Methods

### 2.1. Study Population

Patients with MVA (*n* = 25) characterized by stable effort angina or inducible ischaemia and reduction of the coronary flow reserve, documented by a positive stress test (at least 2.0 mm horizontal or downsloping ST-segment depression) or by a positive SPECT, despite the absence of angiographically documented coronary disease, were recruited. These patients were compared with angiographically documented CAD patients (*n* = 22) and with subjects deemed as healthy on the bases of the absence of clinical symptoms, the instrumental and laboratory examination (Ctrl = 20), and the negative stress test from a previously described cohort  [10]. Exclusion criteria were considered as follows: a history of congestive heart failure, significant valvular diseases, hypertrophic cardiomyopathy, vasospastic angina, recent (<6 months) acute coronary syndrome, surgical or percutaneous revascularization, pacemaker dependency, and atrial fibrillation. Patients with renal insufficiency (serum creatinine concentration >1.4 mg/dL), hepatic disease, recent infection, recent major surgical interventions, immunological disorders, and chronic inflammatory or neoplastic diseases were also excluded. This observational study was carried out in accordance with the Declaration of Helsinki and approved by the local ethics research committee of Centro Cardiologico Monzino (number S1687/610). Written informed consent to participate was obtained from all subjects.

### 2.2. Blood Collection

EDTA-anticoagulated blood was drawn from the antecubital vein of subjects while fasting to obtain whole blood, plasma, and erythrocyte samples. After centrifugation (1,200 g for 10 min at 4°C), plasma was separated and aliquots were stored at −80°C until analyses. Aliquots of packed red cells were lysed by cold deionized water to obtain lysed RBCs and stored at −80°C until analyses.

### 2.3. Arg/NO Metabolic Pathway

We simultaneously measured Arg, ADMA, SDMA, MMA, Cit, and Orn by liquid chromatography-tandem mass spectrometry (LC-MS/MS) [39]. The ratio Arg/(Orn + Cit), as index of global Arg availability [40, 41], and the ratio Orn/Cit, as indicator of the relative activity of arginase and NOS [19], were computed. All the determinations were performed both in plasma and in lysed RBCs.

### 2.4. Oxidative Stress

It was evaluated by the ratio between disulphide and reduced forms of glutathione (GSSG/GSH). GSH and GSSG were measured by LC-MS/MS method on whole blood, after proteins precipitation with trichloroacetic acid [42]. Levels of GSH and GSSG were expressed as *μ*mol/g Hb.

### 2.5. RBC-NOS and Arginase Expression

The RBC-NOS expression was performed by immunofluorescence analysis in a subgroup of subjects (*n* = 10 per group matched for age and sex). RBCs slides were prepared as previously described [10]. Briefly, after blocking of nonspecific reactive sites, RBCs were incubated overnight at 4°C with a monoclonal anti-eNOS (2.5 *μ*g/mL) (BD Biosciences, Milano, Italy) or polyclonal anti-arginase I or monoclonal anti-arginase II (4 *μ*g/mL, for both) (Santa Cruz Biotechnology, DBA Italia s.r.l., Milano, Italy) antibodies. After three washings, an anti-mouse or anti-rabbit AlexaFluor488 conjugated secondary antibody (Invitrogen, Life Technologies Italia, Monza, Italy) was added and the immune complexes were visualized by laser scanning confocal microscope (LSM710, Carl Zeiss, Milano, Italy) using a 63x/1.3 oil immersion objective lens. Images were captured and the fluorescence intensity (densitometric sum of grey) was quantified. Data are expressed as the mean level of fluorescence intensity, subtracted of negative control value obtained on the same slide in the absence of primary antibody. Multiple fields of view (at least three randomly selected areas) were captured for each slide.

### 2.6. Statistics and Scores Development

Numerical variables were summarized as mean and standard deviation (SD), unless otherwise stated, and categorical variables were summarized as frequencies and percentages. A sample size of 20 subjects per group allowed a statistical power of 90% to deem as significant a between-group difference in any analyte approximately equal to one standard deviation, with an alpha error of 0.05. Variables were compared between MVA and CAD or Ctrl by *t*-test or by covariance analysis, adjusting for age and sex. Variables with skewed distribution were log-transformed before analysis. Immunofluorescence intensity was compared between groups by repeated measures covariance analysis, taking into account replicate measures for each subject. All analyses were performed by SAS v. 9.2 (SAS Institute Inc., Cary, NC, USA).

In order to provide a global indicator of all the variables related to NO pathway and to contain inflation of alpha error due to multiple testing, we developed a score similar to the OXY-SCORE, devised by our group few years ago [43]. First, to account for different measurement ranges and units, all the variables were standardized; that is, the mean was subtracted from individual values and the result was divided by the standard deviation. Second, the standardized values of the variables generally accepted as positively associated with endothelial function (Arg and Cit) were added, whereas standardized values of the variables negatively associated with endothelial function (ADMA, SDMA, MMA, and Orn) were subtracted. It is important to note that these associations were intended as “a priori” and were not inferred from the present study. We created a first score using variables measured in plasma (NO plasma score) and another score using variables measured in the RBCs (NO RBC score). Similarly, we created oxidative stress score, a simplified version of the OXY-SCORE including GSSG (with a plus sign) and GSH (with a minus sign).

## 3. Results

### 3.1. Population

The principal demographic and clinical characteristics of the two patient groups and of healthy subjects analyzed in this study are depicted in Table 1. No significant differences were found among groups except for age (*P* = 0.01 MVA versus CAD) that was considered as a confounder for group comparisons.

### 3.2. Biochemical Determinations of Metabolites Involved in Arg/NO Pathway and Oxidative Stress Status

In order to evaluate the potential impairment of Arg/NO pathway in MVA patients, we simultaneously measured the principal metabolites involved in this pathway, both in plasma and in the RBC compartment, and we compared them to the levels measured in CAD and in Ctrl (Table 2). In plasma, MVA patients showed Arg, Cit, and Orn levels similar to those of CAD patients and Ctrl. ADMA levels, instead, were higher in both MVA and CAD patients compared to Ctrl. SDMA and MMA levels did not differ among the three groups studied. In accordance to these findings, the Arg bioavailability (Arg/Orn + Cit ratio) was lower in MVA than in Ctrl and similar to CAD. In addition, the MVA Orn/Cit ratio, an index of activities of the Arg metabolic enzymes arginase and NOS, showed levels intermediate between those of CAD and Ctrl (Table 2).

In the RBC compartment, the levels of NO inhibitors ADMA and SDMA in MVA and CAD patients were higher than in Ctrl (Table 2). Interestingly, MMA levels were the highest in MVA. Arg bioavailability was similar in the three groups of subjects, whereas the Orn/Cit ratio was significantly lower in MVA than in CAD group but similar to Ctrl (Table 2).

**Figure 1:**
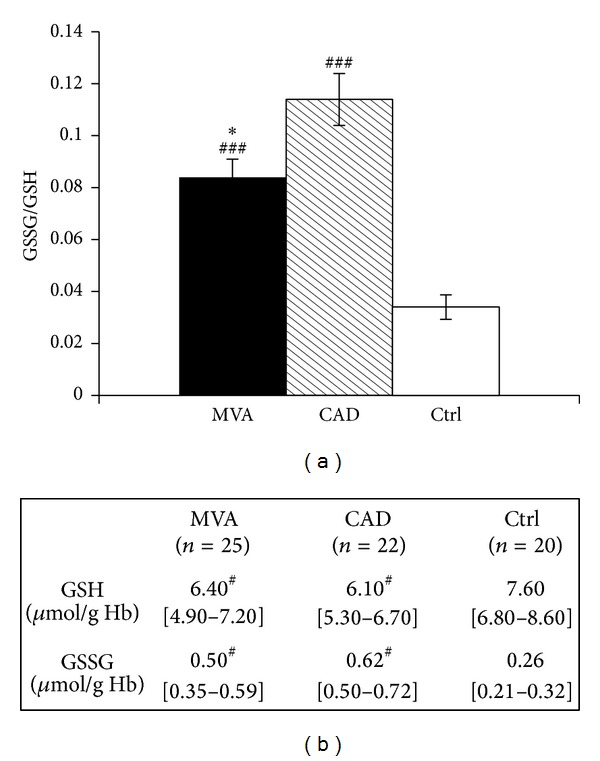
GSSG/GSH ratio in whole blood. The results are expressed as mean ± SE for GSSG/GSH ratio (a) or as median (interquartile range) (b) in whole blood from patients with microvascular angina (MVA *n* = 25) or coronary artery disease (CAD *n* = 22) or healthy subjects (Ctrl *n* = 20). *P < 0.05 versus CAD, ^#^ *P* < 0.05, ^###^ *P* < 0.001 versus Ctrl.

Patients with MVA had higher levels of oxidative stress with respect to Ctrl, but lesser than those determined in CAD patients, as documented by the GSSG/GSH ratio measured in whole blood (Figure 1(a)). Specifically, both groups of patients showed lower levels of GSH and higher levels of GSSG with respect to Ctrl (Figure 1(b)).

**Figure 2:**
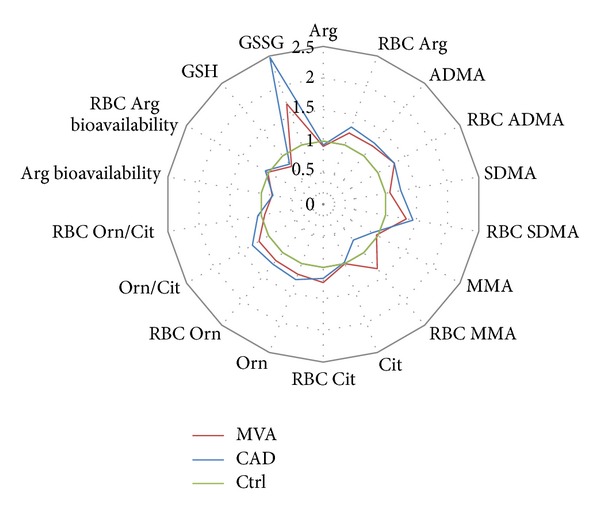
Levels of analytes involved in Arg/NO pathway measured in plasma and in RBCs isolated from patients with microvascular angina (MVA *n* = 25) or coronary artery disease (CAD *n* = 22) or healthy subjects (Ctrl *n* = 20). The results are expressed as fold change over Ctrl.

Figure 2 shows the distribution of the analytes measured in plasma or RBCs of MVA and CAD patients expressed as fold change over Ctrl. In general, the analytes of the NO pathway behaved similarly in MVA and CAD and they were moderately elevated with respect to Ctrl, both in plasma and in RBCs. A special case is represented by MMA in RBCs, whose levels were higher in MVA with respect to Ctrl and CAD patients. As expected, the oxidative stress, in particular the oxidized form of glutathione, was higher in both MVA and CAD patients with respect to Ctrl.

### 3.3. Arginine Metabolic Enzymes: RBC-NOS and Arginase

**Figure 3:**
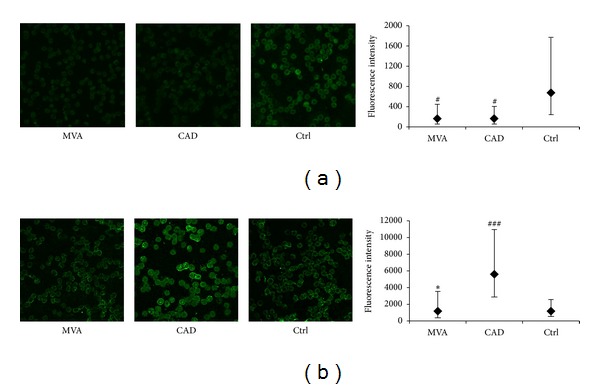
NO synthase and arginase expression in human RBCs. Representative immunofluorescent images (630x magnification) of RBCs isolated from patients with microvascular angina (MVA) or coronary artery disease (CAD) or healthy subjects (Ctrl), stained for RBC-NOS (a) or arginase I (b). Data are expressed as the mean of fluorescent intensity ± SD subtracted of the negative control value (at least three fields were analyzed, *n* = 10 subjects for each group). *P < 0.05 versus CAD; ^#^ *P* < 0.05, ^###^ *P* < 0.001 versus Ctrl.

The expression of RBC-NOS, visualized by immunofluorescence staining, revealed strong quantitative differences between both patient groups and Ctrl. RBCs of MVA and CAD patients had significantly lower RBC-NOS fluorescence, localized in the membrane and into the cytosol, with respect to Ctrl (Figure 3(a)).

The expression of both isoforms of arginase was also evaluated. RBCs of MVA patients and of Ctrl expressed lesser levels of arginase I than CAD patients (*P* = 0.02) (Figure 3(b)). In contrast, the expression of arginase II was not detectable in RBCs of Ctrl and of MVA or CAD patients (data not shown).

### 3.4. Summary Scores of NO Pathway and Oxidative Stress

**Figure 4:**
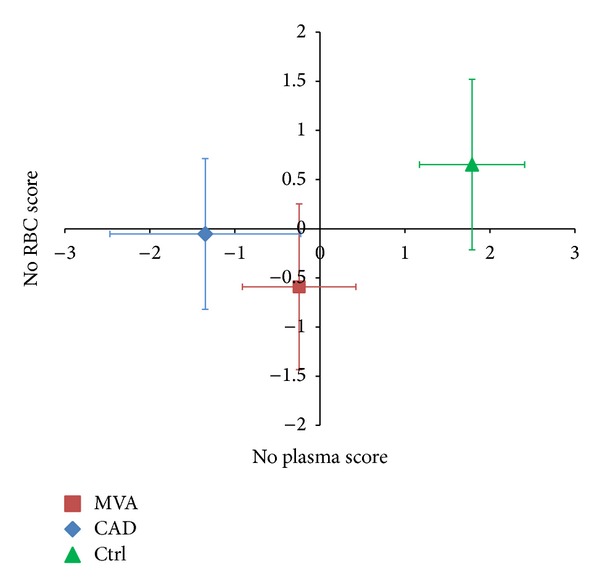
Arg/NO pathway score in plasma and in RBCs. The scores are computed after standardization of the analytes involved in NO synthesis (Arg, Cit, Orn, ADMA, SDMA, and MMA). The standardized values of the variables positively associated with endothelial function are added, whereas the values of variables negatively associated with endothelial function are subtracted. The NO score is calculated in plasma and in RBC compartment. NO RBC score: *P* < 0.05 MVA versus Ctrl; NO plasma score: *P* < 0.001 MVA versus Ctrl and CAD versus Ctrl.

The analytes, that is, substrate, inhibitors, and enzymatic products involved in NO synthesis, were combined into appropriate scores (see Section 2) in order to summarize the Arg/NO pathway in the examined clinical settings. In Figure 4, the Cartesian plane was defined by the NO plasma score (*x*-axis) and the NO RBC score (*y*-axis); the intersection of the axes identifies the midpoint of the entire sample, and the units are expressed in terms of standard deviations. The Ctrl group was placed in the first quadrant (positive values for both scores), whereas the two patient groups were placed in the third quadrant (negative values for both scores). To be noticed, the MVA group was located in a more negative position, along the NO RBC score axis, compared with the CAD group; however, the difference did not reach statistical significance.

**Figure 5:**
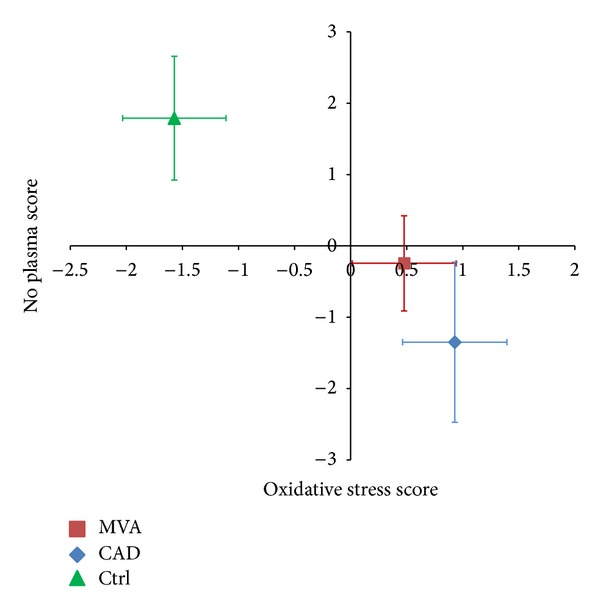
NO plasma and oxidative stress scores. The NO plasma score is computed after standardization of the analytes involved in NO synthesis (Arg, Cit, Orn, ADMA, SDMA, and MMA). The standardized values of the variables positively associated with endothelial function are added, whereas the values of variables negatively associated with endothelial function are subtracted. The oxidative stress score is calculated by the values of GSSG (plus sign) and GSH (minus sign). NO plasma score: *P* < 0.001 MVA versus Ctrl and CAD versus Ctrl; oxidative stress score: *P* < 0.001 MVA versus Ctrl and CAD versus Ctrl.

Figure 5 shows the Cartesian plane defined by the oxidative stress score (*x*-axis) and the NO plasma score (*y*-axis). In this graph, the control group was placed in the quadrant characterized by a negative oxidative score and by a positive NO score. In contrast, both groups of patients were placed in the quadrant relative to a positive oxidative score and a negative NO score, with the CAD group located in a more extreme position with respect to MVA (although this difference did not reach statistical significance: *P* = 0.08 for multivariate ANOVA).

## 4. Discussion

The study described above shows for the first time that RBCs of patients with MVA contain higher levels of inhibitors of the NO synthesis than Ctrl and that these levels do not markedly differ from those found in CAD patients. A similar picture is found in plasma, as previously described by others [37, 38]. Finally, NOS expression in RBCs was found markedly reduced in both MVA and CAD patients. In addition, oxidative stress was found increased in both patient groups, mostly in CAD.

The pathophysiology of MVA is not completely understood yet, even if the several metabolic, haemodynamic, and vasospastic alterations have been linked to this syndrome. Recently, it has been reported that RDW values are significantly higher in both MVA and CAD patients compared to healthy subjects [36]. However, as documented by the absence of modifications in RDW values (data not shown), in our study, the impairment of NO pathway in RBCs of MVA patients is not associated with changes in the size of circulating RBCs. The RBCs of MVA patients, however, showed higher levels of NO synthesis inhibitors and this finding parallels the data found in plasma. As a consequence, in a Cartesian plane, defined by NO scores, the MVA group was located in a negative position along the NO RBC score axis with respect to Ctrl, thus suggesting a possible alteration in NO production, more pronounced in MVA with respect to CAD.

The limitation of our study might be the calculation of the NO scores without measuring NO itself. This highly reactive molecule and its active metabolites are influenced by several factors, including dietary nitrate intake and renal function, particularly in the plasma compartment. Thus, we cannot exclude that other NOS independent factors may add additional information for an overall picture of this metabolic pathway in MVA.

Of interest is the observation that, similar to what previously described for CAD patients [10], we found a marked reduction in NOS expression in RBCs of MVA patients. This finding is of particular relevance because RBCs have a systemic impact in terms of NO production and may represent an important compartment, whose alteration participates to the reduction in the overall NO production.

Arg is the substrate for the NOS enzymes, including RBC-NOS, and it has been shown that an increase of substrate availability in the stenotic lesion induced dilation of the coronary artery segment [44]. Arg is also substrate for the arginase enzyme, whose activity is increased in different pathological conditions associated with a reduction of NO [17, 45].

Two different isoforms of arginase are identified in human and arginase I, which is the only arginase so far described in RBCs, accounts for about 98% of total blood arginase activity [46]. In our condition, greater amounts of arginase I in CAD patients, but not in MVA patients, were found. Since it has been reported that erythroid progenitor cells express both arginase I and arginase II [15], we measured also this enzyme in RBCs. According to the literature [17], we failed to detect measurable amounts of arginase II in Ctrl or in patients.

Increased erythrocyte arginase activity associated with lowered NO plasma levels and with impairment in erythrocytes has been reported in sickle cell disease patients [40]. Interestingly, the consumption of cocoa flavanols reduced the erythrocyte arginase activity, suggesting a possible therapeutic intervention by the regulation of Arg and NO bioavailability [47].

An important condition able to affect NO bioavailability is oxidative stress. Of relevance is the observation that the ratio between oxidized and reduced glutathione was almost doubled in whole blood of MVA patients, suggesting an increased oxidative stress in this condition. A role of oxidative stress in lowering NO bioavailability has been previously highlighted, but the information in MVA is still scanty [27, 48, 49]. We found a marked increase of GSSG/GSH, based on the increase of oxidized glutathione, which was even more pronounced in CAD patients and on a decrease of GSH in MVA patients. This observation is in accordance with data reported by Dhawan et al. [50], who showed a positive correlation between GSH levels and coronary flow velocity reserve, thus predicting impaired microvascular function.

Finally, the concomitant assessment of oxidative stress and NO pathway in patients indicates that both MVA and CAD patients are placed in the Cartesian plane quadrant relative to a positive oxidative score and a negative NO score, with the CAD group located in a more extreme position with respect to MVA.

Thus, as previously suggested by Rassaf and collaborators [51], a multiple-level approach by assessing biochemical, structural, and functional changes in the vasculature may be important for an early diagnosis of cardiovascular diseases and for a better characterization of this multifactorial disease.

## 5. Conclusion

Our study shows that changes in the Arg/NO metabolic profile, coupled to increases in oxidative stress, occur in MVA with a trend toward an impairment similar to that of CAD patients. In particular we have described for the first time alterations in the capacity of RBCs to produce NO in a pathological condition characterized mostly by alterations at the microvascular bed with no significant coronary stenosis.

## Figures and Tables

**Table 1:** Demographic and clinical characteristics of the subjects.

Variable	MVA (*n* = 25)	CAD (*n* = 22)	Ctrl (*n* = 20)
Age (years)	56.5 ± 10.3	66.1 ± 8.6	55.5 ± 10.2
Male gender	14 (56.0)	17 (77.3)	14 (70)
BMI	25.8 ± 3.3	27.3 ± 3.11	24.36 ± 2.35
Total cholesterol (mg/dL)	226.9 ± 59.2	208.3 ± 29.2	208.1 ± 26.8
HDL-cholesterol (mg/dL)	54.9 ± 17.3	46.9 ± 17.2	54.5 ± 17.3
LDL-cholesterol (mg/dL)	150.0 ± 52.4	133.8 ± 36.8	132.3 ± 22.1
Triglycerides (mg/dL)	105.5 ± 66.9	131.5 ± 69.4	95.5 ± 32.5
Systolic blood pressure (mmHg)	130.0 ± 13.2	138.9 ± 18.3	130.0 ± 14.0
Diastolic blood pressure (mmHg)	78.0 ± 8.3	80.0 ± 9.1	78.0 ± 6.0
Creatinine (mg/dL)	0.85 ± 0.22	0.86 ± 0.22	0.81 ± 0.14
Current smoker	3 (12.0)	3 (13.64)	0 (0)
Hypercholesterolemia	14 (56.0)	14 (63.6)	2 (12.5)
Hypertriglyceridemia	1 (4.0)	2 (9.1)	1 (6.25)
Hypertension	11 (44.0)	14 (63.6)	2 (12.5)
*Pharmacological treatments *			
Converting enzyme inhibitors	2 (8.0)	6 (27.3)	0 (0)
Antithrombotics	23 (85.1)	17 (77.3)	0 (0)
Beta-blockers	10 (40.0)	5 (23.8)	1 (5.88)
Calcium channel blockers	1 (4.0)	4 (18.2)	1 (5.88)
Diuretics	2 (8.0)	2 (9.1)	0 (0)
Statins	4 (16.0)	4 (18.2)	2 (11.8)
Hypoglycemics	0 (0)	0 (0)	0 (0)
Angiotensin receptor blockers	4 (16.0)	6 (27.3)	0 (0)

Quantitative variables are expressed as mean ± SD and categorical variables as *n* (%).

**Table 2:** Biochemical determinations in plasma and RBCs.

	Plasma			RBC		
	MVA (*n* = 25)	CAD (*n* = 22)	Ctrl (*n* = 20)	MVA (*n* = 25)	CAD (*n* = 22)	Ctrl (*n* = 20)
Arg	74.22 [69.15–87.08]	82.88 [64.81–95.74]	84.80 [73.13–98.01]	7.57 [5.00–9.53]	8.64 [5.31–10.48]	6.84 [3.86–7.80]
Cit	27.45 [24.21–31.16]	27.61 [20.41–33.83]	26.55 [24.13–30.68]	11.36 [9.58–12.35]	10.48 [8.36–12.11]	8.37 [7.04–10.78]
Orn	47.66 [41.19–54.11]	51.82^#^ [48.72–61.68]	40.84 [34.09–46.08]	39.48 [29.52–49.06]	42.59 [36.74–44.90]	33.93 [23.77–43.07]
ADMA	0.51^#^ [0.43–0.60]	0.49^#^ [0.45–0.59]	0.41 [0.35–0.47]	0.18^#^ [0.14–0.27]	0.20^#^ [0.15–0.25]	0.15 [0.12–0.20]
SDMA	0.47 [0.42–0.60]	0.54 [0.45–0.63]	0.42 [0.36–0.48]	0.13^##^ [0.09–0.16]	0.13^#^ [0.12–0.15]	0.10 [0.06–0.11]
MMA	0.12 [0.09–0.13]	0.11 [0.08–0.13]	0.12 [0.10–0.14]	0.13* [0.07–0.18]	0.05 [0.04–0.09]	0.09 [0.05–0.12]
Arg bioavailability	1.06^#^ [0.90–1.26]	1.01^#^ [0.87–1.22]	1.25 [1.04–1.41]	0.13 [0.11–0.19]	0.16 [0.12–0.19]	0.14 [0.09–0.20]
Orn/Cit ratio	1.74 [1.42–2.21]	1.97^###^ [1.69–2.40]	1.48 [1.36–1.71]	3.88* [3.10–4.41]	3.96 [3.51–5.03]	3.92 [3.20–4.32]

Quantitative variables are expressed as median [interquartile interval]. *P < 0.05 versus CAD; ^#^ *P* < 0.05, ^##^ *P* < 0.01, and ^###^ *P* < 0.001 versus Ctrl adjusted for age and gender after log-transformation of the data.
